# Molecular Conversion of Muscarinic Acetylcholine Receptor M_5_ to Muscarinic Toxin 7 (MT7)-Binding Protein 

**DOI:** 10.3390/toxins3111393

**Published:** 2011-11-11

**Authors:** Sergio Rondinelli, Katja Näreoja, Johnny Näsman

**Affiliations:** Department of Biosciences, Biochemistry, Åbo Akademi University, Tykistökatu 6, FIN-20520 Turku, Finland; Email: srondine@abo.fi (S.R.); kkoivula@abo.fi (K.N.)

**Keywords:** G protein-coupled receptor, muscarinic toxin, acetylcholine receptor, ligand binding

## Abstract

Muscarinic toxin 7 (MT7) is a mamba venom peptide that binds selectively to the M_1_ muscarinic acetylcholine receptor. We have previously shown that the second (ECL2) and third (ECL3) extracellular loops of the M_1_ receptor are critically involved in binding the peptide. In this study we used a mutagenesis approach on the M_5_ subtype of the receptor family to find out if this possesses a similar structural architecture in terms of toxin binding as the M_1_ receptor. An M_5_ receptor construct (M_5_-E^175^Y^184^E^474^), mutated at the formerly deciphered critical residues on ECL2 and 3, gained the ability to bind MT7, but with rather low affinity as determined in a functional assay (apparent *K_i_* = 24 nM; apparent *K_i_* for M_1_ = 0.5 nM). After screening for different domains and residues, we found a specific residue (P^179^ to L in M_5_) in the middle portion of ECL2 that was necessary for high affinity binding of MT7 (M_5_-EL^179^YE, apparent *K_i_* = 0.5 nM). Mutation of P^179^ to A confirmed a role for the leucine side chain in the binding of MT7. Together the results reveal new binding interactions between receptors and the MT7 peptide and strengthen the hypothesis that ECL2 sequence is of utmost importance for MT binding to muscarinic receptors.

## 1. Introduction

Muscarinic acetylcholine receptors (mAChRs) are G protein-coupled receptors that mediate the metabotropic effects of acetylcholine in vertebrates as well as in invertebrates. The mammalian receptor family constitutes five distinct proteins, M_1_-M_5_, with highly homologous sequences and partially overlapping tissue distribution, but with discrete physiological functions [1,2]. At the cellular level, the mAChRs exert their action by activating heterotrimeric guanine nucleotide-binding proteins (G proteins). M_1_, M_3_ and M_5_ receptors are coupled with the G_q_ protein-phospholipase C-Ca^2+^ mobilization pathway, whereas M_2_ and M_4_ are linked to cAMP production and ion channel regulation through G_i_ and G_o_ proteins [1].

Many natural ligands have been found that target the mAChRs. Examples include the agonists muscarine from the fly agaric mushroom and pilocarpine from the *Pilocarpus* plants. The antagonists atropine and scopolamine can be found from the *Solanaceae* plant family. Most of the natural toxins found bind specifically and with relatively high affinity to muscarinic receptors, but they generally lack selectivity among subtypes. Because of the high sequence homology between receptor subtypes, especially in the orthosteric ligand-binding cavity, it has turned out very difficult to chemically produce selective compounds for each subtype [3,4,5].

An unexpected source of subtype-selective muscarinic ligands was found from mamba snakes (genus *Dendroaspis*) [6,7]. These snake venom components are peptide toxins that belong to a large three-finger toxin family most comprehensively characterized by the α-neurotoxins [8,9]. The muscarinic toxins (MTs) are 65-66 amino acids long, contain four disulfide bridges and, because of this structural constraint, form three characteristic loops or fingers protruding out from the disulfide core [10]. The pioneering work on MTs demonstrated that MT1 and MT2 (named after chronological order of discovery) from *D. angusticeps* could not displace all muscarinic receptor ligand bound to rat cortex membranes [6]. This suggested that these MTs can discriminate between subtypes of mAChRs, a fact that later became established using cloned receptor subtypes [11,12]. The MTs found in the venom of *D. angusticeps* seem to target M_1_ and M_4_ receptors while leaving the other subtypes untouched [9]. This might suggest that there is a close structural similarity between the M_1_ and M_4_ subtypes at the toxin-binding site and that the structure for the other subtypes thus deviates from this motif.

Although the MTs show quite high homology (65-80%) among each other, there are also toxins that target more selectively only one receptor subtype. MT3 and MT6 show low nanomolar (1-4 nM) affinities for the M_4_ receptor whereas M_1_ receptor blockade requires a hundredfold (100-300 nM) higher concentrations [9,13,14]. MT7, also called m1-toxin, is unique in the respect that it shows full selectivity for the M_1_ subtype, *i.e.*, subnanomolar affinity for M_1_ and no detectable binding to the other subtypes at micromolar concentrations [15,16]. Since this toxin can discriminate so well among mAChR subtypes, it would be of general interest for ligand-receptor interactions to uncover the binding mechanism for MT7. It has been shown by site-directed mutagenesis of MT7 that many amino acid residues seem to make up the total binding affinity, and these residues are distributed on all three finger loops of the toxin [17]. Regarding the corresponding sites on the receptor, it can be anticipated that binding contacts with loosely conserved regions are crucially involved in the selectivity. Such nonconserved residues are frequently found in the extracellular loops (ECLs) of the receptors. In a previous study, we found that MT7 takes advantage of rather few such nonconserved residues in ECL2 and 3 for discriminating between the M_1_ and M_3_ subtypes [18]. To gain further insight into the structural requirements for MT7 binding, we report in here on the mutagenesis of the M_5_ subtype for gain of binding and functional inhibition effects of the toxin. The results indicate that the structural diversity of receptor ECLs could enable development of subtype-selective compounds using targeting mechanisms employed by the MTs.

## 2. Materials and Methods

### 2.1. Materials

[^3^H]-*N*-methylscopolamine ([^3^H]NMS) (76 Ci/mmol) was purchased from Amersham Biosciences (Buckinghamshire, UK). Fura-2 acetoxymethyl ester was from Molecular Probes (Eugene, OR). Carbamoylcholine chloride (carbachol) and atropine hemisulfate were from Sigma-Aldrich (Helsinki, Finland). Recombinantly expressed MT7 was purified from insect cell medium as described previously [15] and the concentration was determined spectrophotometrically (A_M_ = 15900 M^−1^cm^−1^). Other chemicals used were of analytical grade quality.

### 2.2. Receptor Constructs and Mutagenesis

The human M_1_ and M_5_ receptors have been previously described [19]. All site-directed mutagenesis constructs were generated using the PCR technique with specific oligonucleotide primers with desired mutations (TAG, Copenhagen, Denmark). Subcloning was performed using the pBluescript KS vector (Stratagene, La Jolla, CA) or the pFastBac1 vector (Invitrogen, Paisley, UK) and the correct sequences were verified by automated DNA sequencing (Turku Centre for Biotechnology, Turku, Finland). The chimeric receptor CH1 was a fusion of the M_1_ and M_5_-EYE receptor cDNAs at an ApaI site (nucleotide +474 in M_1_ and nucleotide +489 in M_5_). The chimeric receptor CH2 was a fusion of the M_1_ and M_5_-EYE receptor cDNAs between a StuI site in M_1_ (nucleotide +811) and a PvuII site in M_5_ (nucleotide +1099). All new receptor constructs were finally transferred to the pFastBac1 vector for the generation of recombinant baculoviruses with the Bac-to-Bac baculovirus expression vector system (Invitrogen).

### 2.3. Cell Culture and Receptor Expression

*Spodoptera frugiperda* Sf9 cells were grown in suspension at 27 °C in Grace’s insect medium (Invitrogen, Paisley, UK) supplemented with 8% heat-inactivated fetal bovine serum (Invitrogen), 50 µg/mL streptomycin (Invitrogen), 50 U/mL penicillin (Invitrogen) and 0.02% Pluronic F68 (Sigma-Aldrich, Helsinki, Finland). For recombinant protein expression, Sf9 cells were plated on tissue culture dishes and allowed to attach. A high-titer virus stock was then added to give a multiplicity of infection of 3-5. For the functional assay, the infections were allowed to proceed for 26 h and cultures used for radioligand binding were harvested after 48 h.

### 2.4. Intracellular Ca^2+^ Concentration Measurement

Cells were detached from plates by gentle pipetting, centrifuged 150° *g* for 4 min, resuspended in a smaller volume growth medium and incubated for 20 min with 4 μM fura-2 acetoxymethyl ester at room temperature. Thereafter, cells were centrifuged 150° *g* for 4 min, washed once in assay buffer, resuspended in assay buffer and divided into aliquotes. The aliquotes were briefly spun down in a microcentrifuge, the medium was removed and cell pellets were stored on ice until use. The assay buffer was composed of 130 mM NaCl, 5.4 mM KCl, 1 mM CaCl_2_, 10 mM glucose, 1.2 mM MgCl_2_, 4.2 mM NaHCO_3_, 7.3 mM NaH_2_PO_4_, 63 mM sucrose and 20 mM MES, pH adjusted to 6.3. At the beginning of the experiment, the cells were resuspended in assay buffer with or without MT7 for 5 min. Thereafter the cells were placed in a cuvette in a thermostatted (27 °C) cuvette holder with magnetic stirring in a fluorescence spectrophotometer (Photon Technology International, Ford, UK) and fluorescence recording at alternating 340 nm and 380 nm (excitation) and 510 (emission) was started. Different concentrations of carbachol were added in a cumulative fashion and calibration of each experiment was performed with 0.04% triton X-100 to measure *F*_max_ and 11 mM EGTA to measure *F*_min_. Intracellular free Ca^2+^ concentrations ([Ca^2+^]_i_) were calculated from fluorescence obtained at 340 nm according to: 

[Ca^2+^]_i_ = (*F* − *F*_min_)/(*F*_max_ − *F*) ° 255 nM (*K_d_* for fura-2 − Ca^2+^ complex at 27 °C)     (1)

### 2.5. Radioligand Binding

The Infected cells from monolayer culture were harvested in phosphate-buffered saline solution, centrifuged 700× *g* and stored as pellets at −70 °C until use. For binding experiments the cells were resuspended in binding buffer (100 mM NaCl, 20 mM HEPES, 10 mM MgCl_2_, 1 mM EDTA, pH adjusted to 7.4) and homogenized with an Ultra-Turrax homogenizer. The homogenates (50–100 μg of protein) were incubated with different concentrations (0.01–10 nM) of [^3^H]NMS in a volume of 150 μL for 45 min at room temperature to determine *K_d_* values for the radioligand. Atropine (10 μM) was used to determine nonspecific binding. The inhibition curves for MT7 were obtained by preincubation of homogenates with different concentrations (0.001–300 nM) of MT7 diluted in binding buffer for 45 min at room temperature. Thereafter 0.5 nM [^3^H]NMS was added and incubation continued for another 45 min. The concentrations of MT7 reported were obtained in the final reaction volume. When measuring the radioligand dissociation rate, receptors were first preincubated with 1.5 nM [^3^H]NMS for 30 min. Following this, dissociation was started by addition of 10 μM atropine with or without MT7. Aliquots were removed at regular intervals from 0 to 100 min. In testing the reversibility of MT7 binding, cell homogenates were first incubated with toxin in test tubes for 45 min, thereafter centrifuged 12,000 × *g* for 30 min and then resuspended in fresh buffer. After 15 min the homogenates were added to the binding reactions with 0.5 nM [^3^H]NMS or subjected to another round of centrifugation and resuspension. All reactions were terminated by rapid filtration through prewashed GF/B filters followed by five washes with cold buffer containing 180 mM NaCl, 25 mM MgCl_2_, and 20 mM HEPES, pH 7.4, in a microplate harvester. Radioactivity on filters was determined in a TopCount microplate scintillation counter (PerkinElmer, Waltham, MA). 

### 2.6. Data Analysis

Non-linear curve-fitting of the concentration-response data was performed using GraphPad Prism (GraphPad Software, La Jolla, CA). The pIC_50_ values were obtained through non-linear regression to fit log IC_50_, and the The p*K_x_* values and the cooperativity factor α were obtained through curve-fitting to the allosteric ternary complex model. 

The apparent *K_i_* values in the functional assay were obtained from data fitted to the equation:

Δ[Ca^2+^] = [A]Δ[Ca^2+^]_max_/([A](1 + [I]/*K_i_*') + EC_50_(1 + [I]/*K_i_*))     (2)

[A] and [I] are the concentrations of agonist and inhibitor, respectively, EC_50_ is the [A] producing half-maximal stimulation and *K_i_* and *K_i_*' are the surmountable (competitive) and the insurmountable (noncompetitive) inhibition constants, respectively [20]. The *K_i_* value gives a measure of the right-shift of the concentration-response curve and the *K_i_*' value gives a measure of the suppression of the maximum response. Only the apparent *K_i_* values are given because the apparent *K_i_*' values did not obey inhibitor concentration dependency, as previously also found when using MT7 as inhibitor [18].

## 3. Results and Discussion

### 3.1. Mutagenesis of the M_5_ Receptor Subtype

**Figure 1:**
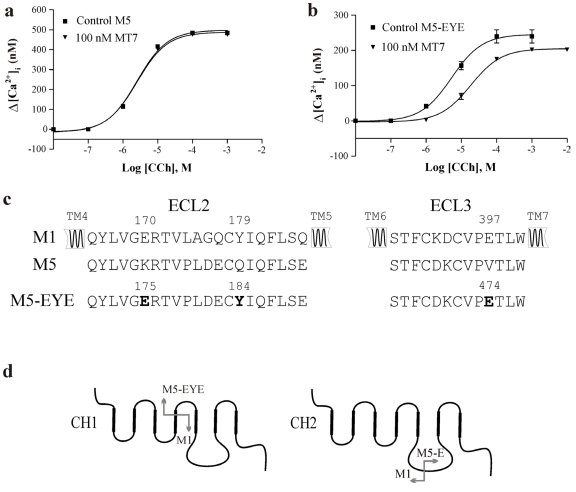
The M_5_ receptor and the M_5_-EYE mutant in Sf9 cells were assayed in the absence and presence of muscarinic toxin 7 (MT7). (**a and b**) Carbachol (CCh) concentration-response curves for [Ca^2+^]_i_ increases. Data points are means ± SD from two experiments; (**c**) Amino acid sequences of extracellular loops ECL2 and ECL3. TM4-7 denotes the helical transmembrane domains of the receptors. Introduced mutations in M_5_ are in boldface; (**d**) Schematic presentation of the chimeric receptors used to localize regions of toxin binding.

**Figure 2:**
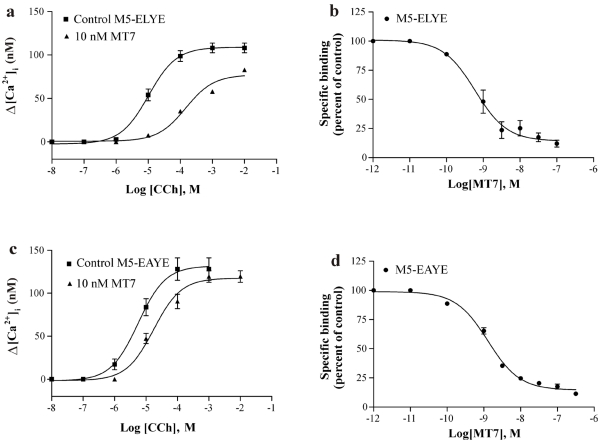
Sf9 cells expressing mutated M_5_ receptor constructs were assayed for carbachol-induced [Ca^2+^]_i_ increases and inhibition of [^3^H]NMS binding. (**a and c**) Functional analyses of the M_5_-ELYE and M_5_-EAYE receptor constructs in the absence and presence of MT7. Data points are means ± SD from two experiments. The apparent *K_i_* values for MT7 were 0.52 ± 0.14 nM and 4.41 ± 1.09 nM (means ± SD, *n* = 4) for M_5_-ELYE and M_5_-EAYE, respectively; (**b and d**) Cell homogenates of each receptor construct were preincubated with different concentrations of MT7 before addition of 0.5 nM [^3^H]NMS. Bound radioactivity was converted to receptor concentration and presented as percent of control values in the absence of toxin. Data points are means ± SEM from three experiments.

Functional inhibition of receptors by MT7 was assessed by measuring the [Ca^2+^]_i_ responses in Sf9 cells expressing intact or modified receptor proteins. Activation of the M_5_ receptor with carbachol resulted in concentration-dependent increases in [Ca^2+^]_i_ as determined from fura-2 fluorescence (Figure 1a). MT7 did not affect the activation of the intact M_5_ subtype. To try to gain binding of MT7, we introduced two mutations in ECL2 and one in ECL3 of M_5_ (M_5_-EYE, Figure 1c). The glutamate residue in the proximal part of ECL2 of M_1_ (E^170^ in M_1_, K^175^ in M_5_) and a tyrosine residue adjacent to the conserved cysteine (Y^179^ in M_1_, Q^184^ in M_5_) were both previously recognized as important for MT7 binding [18]. These residues together with the V^474^ to E mutation in ECL3 rendered the M_5_ subtype sensitive to MT7 (Figure 1b). The apparent *K_i_* (>20 nM, Table 1) was, however, significantly higher than what we obtained with the M_1_ subtype (apparent *K_i_* = 0.45 ± 0.18 nM, mean ± SD, *n* = 3). This prompted us to look for additional interaction sites between the toxin and the receptor. First we used chimeric M_1_:M_5_-EYE receptors to locate the region involved (Figure 1d). These chimeras (CH1 and CH2) indicated that we should refocus on the ECL2, since CH2 incorporating the ECL2 of M_1_ increased the affinity for MT7 about tenfold as compared to M_5_-EYE (Table 1). After screening for individual residues in ECL2 of M_5_, we found that the additional P^179^ to L (M_5_-ELYE) mutation increased significantly the affinity for MT7 (Figure 2a). This result was further confirmed in radioligand binding experiments with a calculated IC_50_ of 0.6 nM for MT7 (Figure 2b, Table 2). As for the M_1_ subtype (IC_50_ ≈ 0.5 nM) [18,21], MT7 does not compete directly with the orthosteric antagonist and a fraction of the radioligand binding remains even at saturating MT7 concentrations. Thus, the potencies for inhibition are presented as pIC_50_ values and not converted to *K_i_* values as for competitive ligands (Table 2).

**Table 1:** Functional characteristics of expressed receptor constructs. Values are means ± SD from three experiments.

Construct	EC_50_ for CCh, μM	Max. Δ[Ca^2+^]_i_, nM	App. *K_i_* for MT7, nM
M_5_	2.56 ± 0.34	498 ± 10	−
M_5_-EYE	5.29 ± 0.03	246 ± 33	24.06 ± 4.44
CH1	4.60 ± 1.61	236 ± 10	11.54 ± 2.56
CH2	6.14 ± 1.10	293 ± 17	1.85 ± 0.61

**Table 2:** Binding characteristics of expressed receptor constructs. Values are means ± SEM from three experiments.

Construct	*K_d_* for [^3^H]NMS, nM	pIC_50_ for MT7	p *K_x_* for MT7	Cooperativity factor α
M_5_	0.79 ± 0.16	−	−	−
M_5_-ELYE	0.54 ± 0.09	9.22 ± 0.11	9.48 ± 0.10	0.08 ± 0.02
M_5_-EAYE	0.49 ± 0.08	8.89 ± 0.04	9.16 ± 0.04	0.08 ± 0.01
M_5_-ELY	0.73 ± 0.09	8.21 ± 0.05	8.38 ± 0.04	0.21 ± 0.01

The P^179^t to L mutation in the middle of ECL2 of the M_5_ receptor appeared to have an important role in the binding of MT7. The corresponding residue of the M_1_ receptor we did not specifically investigate in our previous study, and the M_3_ double mutant with the corresponding P^217^ to L and the additional P^218^ to A mutations did not show an inhibitory effect of MT7 [18]. To further investigate whether the aliphatic side chain of the leucine is important for the binding or if the proline only postures a steric hindrance for other interactions to take place, we mutated the P179 to A and analyzed the receptor construct in the functional assay (Figure 2c) and with radioligand binding (Figure 2d, Table 2). MT7 was found to possess lower potency for M_5_-EAYE in both assays when compared to M_5_-ELYE, indicating a contribution to the overall binding for the leucine side chain.

### 3.2. The Role of the ECL3 Glutamate

**Figure 3:**
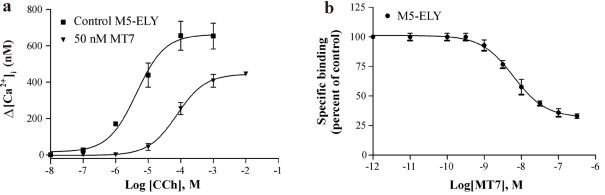
Sf9 cells expressing the M_5_-ELY construct were assayed for carbachol-induced [Ca^2+^]_i_ increases and inhibition of [^3^H]NMS binding. (**a**) Representative concentration-dependent increases in [Ca^2+^]_i_ for control and in the presence of 50 nM MT7. The apparent *K_i_* was determined to 2.06 ± 0.86 nM (mean ± SD, *n* = 3); (**b**) Titration of MT7 against [^3^H]NMS with M_5_-ELY. Data points are means ± SEM from three experiments.

In our previous study using chimeric M_1_:M_3_ receptors, we found that a lysine residue (K^523^) in M_3_ ECL3 sequence resulted in a tenfold loss in the apparent *K_i_* value for MT7 [18]. It was not clear, however, whether this was due to a loss of binding contact with the corresponding negatively charged glutamate (E^397^) of M_1_ or if the long acyl chain and positively charged head group of the M_3_ lysine somehow precluded other types of interactions between the receptor and the toxin. In the M_5_ receptor, the corresponding residue is a valine (V^474^), which should at least not exhibit any charge repulsion. We thus exchanged the mutated ECL3 of M_5_-ELYE for the intact M_5_ sequence and measured the binding characteristics of MT7 (Figure 3). In the functional assay, the M_5_-ELY construct lost an approximately fourfold blocking potency (apparent *K_i_* = 2.06 ± 0.86 nM, mean ± SD, *n* = 3) as compared to M_5_-ELYE, and about tenfold loss in binding affinity (Table 2). In addition to the loss in binding affinity, we noticed that the inhibitory capacity of MT7 in terms of displacement of radioligand was significantly lower than for the other receptor constructs, *i.e.*, the remaining bound radioligand in the presence of saturating concentrations of MT7 mounted to about 30% for M_5_-ELY (Figure 3b), whereas for M_5_-ELYE (Figure 2b) and for M_1_ the corresponding level was about 15%. We do not currently have an explanation for these different behaviors.

### 3.3. Allosteric Interactions and Irreversibility of Binding

**Figure 4:**
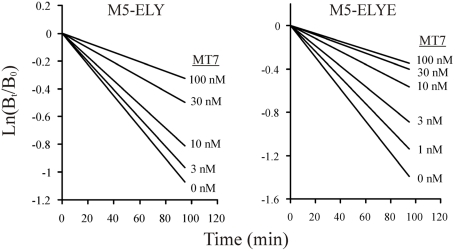
Deceleration of [^3^H]NMS dissociation by MT7. The M_5_-ELY and M_5_-ELYE constructs were preincubated with [^3^H]NMS and dissociation initiated by addition of atropine with or without different concentrations of MT7. Samples were collected at different time points and [^3^H]NMS binding (B_t_) determined. Data presented are from one experiment performed with triplicate samples. Two additional experiments with 10 and 100 nM MT7 gave similar results. Only the trendlines are shown for clarity. The control rate for M_5_-ELY was 0.113 min^−1^ and 0.003 min^−1^ with 100 nM MT7. The control rate for M_5_-ELYE was 0.147 min^−1^ and 0.004 min^−1^ with 100 nM MT7.

**Figure 5:**
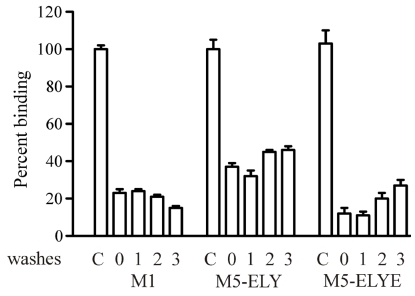
Reversibility test for MT7. Receptor constructs were preincubated with MT7 (30 nM with M_1_ and M_5_-ELYE, 100 nM with M_5_-ELY) for 45 min and then subjected to [^3^H]NMS binding (control (C) and zero washes) or centrifuged and washed one to three times before [^3^H]NMS binding. Data are means ± SEM from three experiments.

In addition to the high affinity binding of MT7 to the M_1_ receptor, the characteristics of this toxin−receptor complex also include an allosteric slowing of antagonist dissociation [22,23], and a pseudo-irreversibility of binding, *i.e.*, the toxin does not dissociate from the receptor in spite of extensive washing [7,24]. To find out if our M_5_ constructs exhibited similar properties, we first tested the ability to slow down the dissociation of prebound [^3^H]NMS. With both M_5_-ELY and M_5_-ELYE there was a concentration-dependent deceleration of antagonist dissociation (Figure 4). The pIC_50,diss_ values (affinity for [^3^H]NMS-occupied receptor) were 7.73 ± 0.06 and 8.47 ± 0.06 for M_5_-ELY and M_5_-ELYE, respectively. We also tested the irreversibility of MT7 binding at M_1_ and the mutated M_5_ constructs. We have previously used a protocol with repeated centrifugations to demonstrate the reversibility of MTα binding to the α_2B_ adrenergic receptor [25]. Using the same protocol for MT7, we could not recover control binding sites with either M_1_ or the mutated M_5_ constructs (Figure 5). These data indicate that the M_5_ constructs have been converted to M_1_ phenotypes in the known pharmacological aspects of MT7 binding.

## 4. Conclusions

The MT7 toxin shows an astonishing selectivity for the M_1_ receptor, making it an attractive template for how to develop receptor subtype-selective compounds. To accomplish such a task, we need to understand how MT7 binds to the receptor and which receptor epitopes are chosen by the toxin. We have in this study converted by mutagenesis the M_5_ receptor to bind MT7 with high affinity, without interfering to any larger extent with orthosteric antagonist binding (as determined from measured affinities of [^3^H]NMS) or agonist activation of the receptors (as determined from the functional assays). The importance of the leucine residue found in the middle of ECL2 of the M_1_ receptor expands the list of critical residues involved in the binding of MT7.

During the preparation of this manuscript, Marquer *et al*. reported on the mutation of the M_1_ receptor and MT7. They also identified the leucine in the middle of ECL2 as a player in the toxin interactions [26]. Mutation of the leucine to proline, as in M_3_, decreased the binding affinity for MT7. On the other hand, the leucine to alanine mutation did not appear to affect toxin binding. This is in contrast to what we found with the M_5_ receptor. It is possible that MT7 binds with slightly altered conformation to the mutated M_5_ receptor than to the M_1_ receptor and thus utilizes receptor residues that have a less important role in M_1_. It may also be that our approach to mutate other receptor subtypes for gain of binding reveal more subtle interactions if there are less overall interaction sites as compared to the MT7−M_1_ receptor complex.

## References

[1] Caulfield M.P., Birdsall N.J. (1998). International Union of Pharmacology. XVII. Classification of muscarinic acetylcholine receptors. Pharmacol. Rev..

[2] Wess J. (2004). Muscarinic acetylcholine receptor knockout mice: Novel phenotypes and clinical implications. Annu. Rev. Pharmacol. Toxicol..

[3] Eglen R.M., Choppin A., Dillon M.P., Hegde S. (1999). Muscarinic receptor ligands and their therapeutic potential. Curr. Opin. Chem. Biol..

[4] Eglen R.M., Choppin A., Watson N. (2001). Therapeutic opportunities from muscarinic receptor research. Trends Pharmacol. Sci..

[5] Heinrich J.N., Butera J.A., Carrick T., Kramer A., Kowal D., Lock T., Marquis K.L., Pausch M.H., Popiolek M., Sun S.C., Tseng E., Uveges A.J., Mayer S.C. (2009). Pharmacological comparison of muscarinic ligands: Historical versus more recent muscarinic M1-preferring receptor agonists. Eur. J. Pharmacol..

[6] Adem A., Åsblom A., Johansson G., Mbugua P.M., Karlsson E. (1988). Toxins from the venom of the green mamba *Dendroaspis angusticeps* that inhibit the binding of quinuclidinyl benzilate to muscarinic acetylcholine receptors. Biochim. Biophys. Acta.

[7] Max S.I., Liang J.S., Potter L.T. (1993). Purification and properties of m1-toxin, a specific antagonist of m1 muscarinic receptors. J. Neurosci..

[8] Tsetlin V. (1999). Snake venom alpha-neurotoxins and other “three-finger” proteins. Eur. J. Biochem..

[9] Karlsson E., Jolkkonen M., Mulugeta E., Onali P., Adem A. (2000). Snake toxins with high selectivity for subtypes of muscarinic acetylcholine receptors. Biochimie.

[10] Ségalas I., Roumestand C., Zinn-Justin S., Gilquin B., Ménez R., Ménez A., Toma F. (1995). Solution structure of a green mamba toxin that activates muscarinic acetylcholine receptors, as studied by nuclear magnetic resonance and molecular modeling. Biochemistry.

[11] Kornisiuk E., Jerusalinsky D., Cervenansky C., Harvey A.L. (1995). Binding of muscarinic toxins MTx1 and MTx2 from the venom of the green mamba *Dendroaspis angusticeps* to cloned human muscarinic cholinoceptors. Toxicon.

[12] Kornisiuk E., Jerusalinsky D., Cervenansky C., Harvey A.L. (1995). Corrigendum. Toxicon.

[13] Jolkkonen M., van Giersbergen P.L., Hellman U., Wernstedt C., Karlsson E. (1994). A toxin from the green mamba *Dendroaspis angusticeps*: Amino acid sequence and selectivity for muscarinic m4 receptors. FEBS Lett..

[14] Liang J.S., Carsi-Gabrenas J., Krajewski J.L., McCafferty J.M., Purkerson S.L., Santiago M.P., Strauss W.L., Valentine H.H., Potter L.T. (1996). Anti-muscarinic toxins from *Dendroaspis angusticeps*. Toxicon.

[15] Näsman J., Jolkkonen M., Ammoun S., Karlsson E., Åkerman K.E.  (2000). Recombinant expression of a selective blocker of M(1) muscarinic receptors. Biochem. Biophys. Res. Commun..

[16] Mourier G., Dutertre S., Fruchart-Gaillard C., Menez A., Servent D. (2003). Chemical synthesis of MT1 and MT7 muscarinic toxins: Critical role of Arg-34 in their interaction with M1 muscarinic receptor. Mol. Pharmacol..

[17] Fruchart-Gaillard C., Mourier G., Marquer C., Stura E., Birdsall N.J., Servent D. (2008). Different interactions between MT7 toxin and the human muscarinic M1 receptor in its free and *N*-methylscopolamine-occupied states. Mol. Pharmacol..

[18] Kukkonen A., Peräkylä M., Åkerman K.E., Näsman J. (2004). Muscarinic toxin 7 selectivity is dictated by extracellular receptor loops. J. Biol. Chem..

[19] Kukkonen J.P., Näsman J., Ojala P., Oker-Blom C., Åkerman K.E.O.  (1996). Functional properties of muscarinic receptor subtypes Hm1, Hm3 and Hm5 expressed in Sf9 cells using the baculovirus expression system. J. Pharmacol. Exp. Ther..

[20] Kukkonen J., Åkerman K.E. (1992). Apparent noncompetitive antagonism of muscarinic receptor mediated Ca^2+^ mobilization by some muscarinic antagonists. Biochem. Biophys. Res. Commun..

[21] Näreoja K., Kukkonen J., Rondinelli S., Toivola D., Meriluoto J., Näsman J. (2011). Adrenoceptor activity of muscarinic toxins identified from mamba venoms. Br. J. Pharmacol..

[22] Max S.I., Liang J.S., Potter L.T. (1993). Stable allosteric binding of m1-toxin to m1 muscarinic receptors. Mol. Pharmacol..

[23] Fruchart-Gaillard C., Mourier G., Marquer C., Menez A., Servent D. (2006). Identification of various allosteric interaction sites on M1 muscarinic receptor using 125I-Met35-oxidized muscarinic toxin 7. Mol. Pharmacol..

[24] Olianas M.C., Maullu C., Adem A., Mulugeta E., Karlsson E., Onali P. (2000). Inhibition of acetylcholine muscarinic M(1) receptor function by the M(1)-selective ligand muscarinic toxin 7 (MT-7). Br. J. Pharmacol..

[25] Koivula K., Rondinelli S., Näsman J. (2010). The three-finger toxin MTalpha is a selective alpha(2B)-adrenoceptor antagonist. Toxicon.

[26] Marquer C., Fruchart-Gaillard C., Letellier G., Marcon E., Mourier G., Zinn-Justin S., Menez A., Servent D., Gilquin B. (2011). Structural model of ligand-G protein-coupled receptor (GPCR) complex based on experimental double mutant cycle data: MT7 snake toxin bound to dimeric hM1 muscarinic receptor. J. Biol. Chem..

